# Openness in Big Data and Data Repositories: The Application of an Ethics Framework for Big Data in Health and Research

**DOI:** 10.1007/s41649-019-00097-z

**Published:** 2019-10-01

**Authors:** Vicki Xafis, Markus K. Labude

**Affiliations:** Centre for Biomedical Ethics, Yong Loo Lin School of Medicine, National University of Singapore, Singapore

**Keywords:** Big data, Open data, Open science, Data repository, Decision-making framework, Health data

## Abstract

There is a growing expectation, or even requirement, for researchers to deposit a variety of research data in data repositories as a condition of funding or publication. This expectation recognizes the enormous benefits of data collected and created for research purposes being made available for secondary uses, as open science gains increasing support. This is particularly so in the context of big data, especially where health data is involved. There are, however, also challenges relating to the collection, storage, and re-use of research data. This paper gives a brief overview of the landscape of data sharing via data repositories and discusses some of the key ethical issues raised by the sharing of health-related research data, including expectations of privacy and confidentiality, the transparency of repository governance structures, access restrictions, as well as data ownership and the fair attribution of credit. To consider these issues and the values that are pertinent, the paper applies the deliberative balancing approach articulated in the *Ethics Framework for Big Data in Health and Research* (Xafis et al. 2019) to the domain of Openness in Big Data and Data Repositories. Please refer to that article for more information on how this framework is to be used, including a full explanation of the key values involved and the balancing approach used in the case study at the end.

## Background

“Openness” in scientific research relates to the sharing, in a usable way, of scholarly publications and data resulting from scholarly research (including metadata and the methodology, as well as codes or algorithms that were used to generate the research data shared). This paper examines some of the ethical considerations that arise with the sharing of data through online data repositories in health and biomedical research. Data repositories represent *only one* mode of data sharing; other modes may include posting data on institutional or researchers’ websites, providing data to requestors personally, and making data accessible through publications (e.g. through supplementary files).

Sharing data through well-curated online data repositories presents opportunities as well as challenges. For example, a distinct advantage is that online data repositories create a central “pool” of data and make the data easily discoverable for bona fide researchers worldwide to access and re-use. Ideally, the storage of data in research data repositories also ensures the long-term availability of data beyond the end of a particular research project. A corresponding challenge concerns questions about the appropriate governance mechanisms for data repositories, including questions about *who* will be able to access the data and *what* (if any) levels of restriction should be applied. Another practical yet pervasive challenge is researchers’ ability to make use of data in data repositories. This could be due to the quality of the data, its formatting, or the absence of metadata. The *FAIR Data Principles* reflect the features that must characterise data and other research products so that humans and, importantly, machines can fully understand and use them (Wilkinson et al. 2016). To be of value, according to the FAIR principles, data must be: Findable, Accessible, Interoperable, and Re-usable (Wilkinson et al. 2016).

The proliferation of data sharing policies, practices, and mandates has occurred over a number of years. Despite this, there is evidence to suggest that researchers have not kept up with these developments; researchers continue to display limited understanding of data sharing environments, including knowledge of repositories and issues such as copyright and licensing (Stuart et al. 2018).

### Types of Data Repositories

Data repositories are not uniform. They differ in terms of who holds the data as well as the nature of the data held and could often be considered to belong to more than one of the categories below.

*Institutional Data Repositories*: These repositories are often university-based. They manage and disseminate the research output (primary facts and statistics but also source codes and developed software tools) generated by members of an institution’s own research community. A good example is the University of Bristol’s Research Data Service has developed a central repository with accompanying governance, technical, and workflow structures that enhance the responsible sharing of data (Merrett et al. 2018). The management and dissemination of institutional data is also supported by web-based repositories such as Figshare (https://figshare.com).

*Government Data Repositories*: Governments hold vast amounts of data routinely collected for administrative purposes, health surveillance, and the delivery and management of healthcare. The value of access to such data for health and biomedical research is increasingly being recognised by governments and the research community. The tension between sharing such data and concerns about privacy protections remains a central issue but increasingly there are governance solutions to facilitate the re-use of valuable government data sets (Ubaldi 2013).

*Discipline-Specific Data Repositories*: Discipline-specific data repositories contain data and metadata pertaining to specific subject areas, such as health sciences or earth and environmental sciences. Such repositories are valuable because they provide a single point for discipline-specific data discovery and retrieval. They are also necessary as domain-specific software is often required to convert file formats of data in various disciplines.

*Generalist Data Repositories*: Generalist data repositories are suitable for the deposition of data where no discipline-specific repository exists. *Scientific Data* advises that such repositories are also suitable “for archiving associated analyses, or experimental-control data, supplementing the primary data in a data-type specific repository” (Scientific Data n.d.).

*Project/Program-specific Repositories*: Program/project-specific repositories comprise collections of data collected as part of a specific body of research. An example of such a repository is the repository for the *Growing Up Today Study*, whose aim is to collect data from thousands of participants to investigate factors that affect health throughout life (https://www.re3data.org/repository/r3d100011832).

### Support for Data Sharing

There is general support for data sharing from numerous stakeholders. This includes the scientific community, through international bodies, such as the *International Council for Science* (International Council for Science (ICSU) 2015), and funding bodies, such as the National Institutes of Health (U.S. Dept of Health and Human Services 2018), the European Commission (European Commission 2012), and the Australian Research Council (Australian Research Council 2018). Funding bodies either mandate or encourage grantees to submit a data management plan detailing how research outputs will be shared. Perhaps most importantly, many academic journals increasingly require researchers to make underlying data available upon scholarly publication (Taichman et al. 2016; Federer et al. 2018). The *Transparency and Openness Promotion* (TOP) *Guidelines* developed by the *Center for Open Science* articulate three levels of transparency, each requiring greater commitment to open sharing, and these have been adopted by journals in most fields and increasingly by funding bodies around the world (Nosek et al. 2015). These and other stakeholders articulate the benefits in support of open data sharing shown in Table 1.

**Table 1:** Benefits of data sharing

Research integrity and the promotion of scientific rigour	
• Reproducibility and replicability for independent verification of research results • Detection of research errors and fraudulent research	
Public benefit and harm minimisation	
• Increase in the utility of existing datasets • Responsible use of public funds • Safer and better informed clinical practice and policy implementation • Reduction of the research burden on frequently studied individuals/groups • Data preservation	
Personal and professional benefits	
• Opportunities for cross-domain, collaborative research • Reduction of duplication of effort and cost considerations • Validation of findings and building on published work	

### Data Sharing Attitudes on the Ground

Researchers’ attitudes toward data sharing appear to be influenced by discipline and discipline-specific normative pressures (i.e. established norms within their disciplines) but not by funding agency mandates, perhaps because of the lack of checks and penalties (Tenopir et al. 2015).

Conversely, pressure to conform to open data practices and incentivisation from scientific journals appear to increase researchers’ data sharing practices. Researchers engaging with human subjects, such as those in health and medicine, are less likely to engage in data sharing, as many believe they do not have the right to share the data or are unsure about copyright and licensing (Tenopir et al. 2015). Furthermore, making data accessible and developing the required metadata is time-consuming and the perceived effort to achieve this also acts as a deterrent to data sharing (Stuart et al. 2018).

There appear to also be some age-related differences between the perception about the value of data sharing and the actual data sharing practices: older researchers (50+) claim to share significantly more data than younger researchers but younger researchers indicate a more positive outlook on data sharing (Tenopir et al. 2015).

### Degrees of Openness

Although there is a general ambition in the scientific community to strive for a model of Open Data sharing, ethical considerations sometimes call for access restrictions where human subject data is concerned, especially in the health and biomedical sciences (Merrett et al. 2018; Boulton et al. 2012). A key consideration here is whether the data that is to be shared consists of aggregate research data or of individual participant data (IPD). The sharing of IPD, even if de-identified, may give rise to re-identification concerns in the context of big data. In contrast, the sharing of aggregate data would generally not disclose information about individuals and, hence, would be safer to share openly. However, aggregate research data does not always allow for full reproducibility of results and is less beneficial for future research use (see for example Huang et al. 2016).

The different models of access restriction vary significantly (Lowrance 2012) as do specific definitions but, generally, data access levels fall somewhere into the broad spectrum of *open*, *restricted*, and *controlled.* These access levels have been developed with two disparate mechanisms in mind: (1) security mechanisms to ensure that only bona fide researchers bound by professional obligations and specific agreements have access to the data under certain data security conditions; and (2) participant consent. Consent does not provide protections against potential re-identification but does enable the research participant to assume or decline to assume potential risks associated with access to their de-identified data.

- Open Data^1^ comes with (almost) no access restrictions;
- Restricted Data requires some level of clearance before access is granted. Restricted Data may include IPD where consent has not been granted for open sharing but where re-identification is considered a low risk;
- Controlled Data carries a higher risk of re-identification and cannot be openly shared as consent is not available (Merrett et al. 2018). Decisions regarding access are invariably made by Data Access Committees (DACs) rather than a single individual.

There is a wide variety of data security mechanisms deployed by repositories, often linked to the sensitivity of the data. Examples of security mechanisms include, but are not limited to, the following: various levels of control may be imposed by the repository developer and custodian often through formal data sharing agreements with explicit researcher and institutional obligations articulated, including a mandate not to attempt to re-identify participant data; data may be shared over secure platforms and may not be downloadable; there are sometimes requirements for members of the data repository to collaborate on projects; with some data, there are audit trails to provide greater accountability and protections.

## Key Issues

Funding bodies, publishers, and governments alike are strong supporters of open data sharing (and consequently the use of trusted repositories), but there are several issues requiring consideration. The following is not an exhaustive list:

- Privacy and Confidentiality^2^: Traditional data protection models of anonymising or de-identifying data have been criticised for no longer guaranteeing protection against the re-identification of research participants (Ohm 2010). The sheer amount of data available and increased technical capabilities may facilitate the re-identification of individuals (see example on Genomic Data Sharing in textbox). Concerns about re-identification may be especially pronounced for individuals’ health and medical data, which tends to be considered “sensitive data”.^3^
- Access Restrictions and Transparent Governance: Much of the data that is generated and used in health and medical research would be considered “sensitive data” (Merrett et al. 2018) and may be de-identified yet carry a re-identification risk. A key question is how to regulate access to such research materials in a way that allows beneficial data sharing on a world stage to take place while protecting the rights and welfare of the participants to whom the data pertains.
  Legal provisions may partly dictate how to evaluate such access requests particularly if they originate from a foreign source. Specifically, sensitive data may be transferred to a foreign jurisdiction only if there are satisfactory or equivalent levels of data protection in the receiving jurisdiction (OECD 2015). This, however, may prevent researchers from countries with less developed data protection regimes from accessing datasets.
  Another concern relating to access restrictions in data repositories is that governance and decision-making mechanisms may not be transparent to those who seek access. For example, it may be unclear what the data access criteria are, who is making determinations about the appropriateness of data access, and what justifications support rejections for access.
- Data Ownership and the Fair Attribution of Credit: Even though funders and publishers tend to provide strong mandates for data sharing, several factors may help explain why researchers remain reticent to deposit data in (open) repositories (Tsoukala et al. 2015). One factor relates to beliefs about data ownership: a global survey of 1200 researchers suggests that a sizable proportion (47%) believe they retain ownership over the data that they have generated in their research, even after publication (Berghmans et al. 2017). This perception of ownership may, in turn, ground a reluctance to share data via repositories, where researchers may no longer have full control over who has access to “their” data. The same survey also revealed that researchers hold (mistaken) beliefs about ownership of data in published research papers with 38% believing that publishers gain ownership of data after the publication of academic papers (Berghmans et al. 2017). Accordingly, researchers may no longer feel confident that they are in a position to openly share the data that underlie the publication.
  A related factor cited as a barrier to the sharing of data in research data repositories concerns insufficient attribution (Longo and Drazen 2016). While it appears to be widely accepted that those who make data available for others to use *should* receive credit or acknowledgement (Tenopir et al. 2015), researchers remain concerned that there are no clear standards for citing others’ data and that, even if data is cited, there is lack of professional credit for having made the data available (Berghmans et al. 2017). As such, the push toward increased data sharing practices would benefit from establishing clear standards of attribution and from a better alignment of professional evaluation structures with current data sharing expectations.

Aiming to assist with the identification of uptake issues and their resolution via policy recommendations on open access to research data is the project on *Policy RECommendations for Open Access to Research Data in Europe* (Tsoukala et al. 2016)*.* Valuable guidance is provided for the different stakeholders (e.g. funders, publishers, data managers, research institutions) in recognition of the different roles they play in the open access ecosystem (Tsoukala et al. 2015). Additional issues, such as access by commercial actors to publicly funded research data, are also addressed (Finn et al. 2014).

### Conflicts in Guidance and Policies

Data sharing is currently at various levels of implementation across the research spectrum worldwide. Hence, it may be mandated, encouraged, or not yet considered systematically in any phase of the research cycle including in the development of research proposals where such issues should be considered. As a result, the data sharing requirements of various entities may clash. For example, a scientific journal may mandate deposition and sharing of all research materials and products but a university may not yet formally consider data sharing as standard practice. On the other hand, Institutional Review Boards (IRBs) and other areas of the university are likely to have restrictive policies in relation to the disclosure and sharing of participant level data. Differences in risks associated with data sharing also arise from the different kinds of data shared, as the following example illustrates.

**Table:** 

**Example: Genomic data sharing** The sharing of genomic data generates particularly difficult issues around privacy and confidentiality. Genomic data has been shared for a number of years backed by healthcare professionals and citizens who have advocated for its open use and re-use (Topol 2015). Some of the considerations in genomic research include the following: 1. When genomic data is shared openly, it can never be withdrawn from the public sphere, and it is impossible to know the uses to which it might be put (Heeney et al. 2011). Inability to remove data from public access also means that it is impossible for research participants to withdraw completely from current and future research. This is evidently an important consideration in the consent process and participants must bear the “costs” if they later have a change of heart regarding participation. 2. Re-identification of participants from their genomic data is increasingly possible (Erlich et al. 2018). It is, therefore, important not to create expectations of lasting anonymity to participants who consent to the open sharing of their genomic data. 3. It is not possible for researchers to foresee the kinds of uses to which data will be put in the future with some uses potentially leading to re-identification which could result in harms. Likewise, public benefits/harms are not entirely anticipatable, which may pose a challenge to meeting participants’ expectations of social benefits that motivates their willingness to make available their genomic data. 4. Although data is often shared globally, legislative protections are not uniform across jurisdictions (e.g. anti-discrimination legislation). The difference in legal protections afforded can impact significantly not only on individuals but also on families and entire ethnic groups. Researchers need to bear such broader considerations in mind when engaging participants. 5. Concerns have also been raised about the discrepancy between the privacy protections afforded to participants and those afforded to biological relatives whose data is inevitably also shared but whose consent is not sought (Takashima et al. 2018). In view of the specific ethical issues raised in genomic and other kinds of research where sensitive health data is used, familiarity with the licence agreements and the conditions of access is essential, as is an understanding that researchers depositing data can impose conditions on access and re-use to protect participants’ confidentiality and privacy (Mauthner and Parry 2013).	

## Key Values

Many of the substantive and procedural values in this Framework (Xafis et al. 2019) bear on the practice of data sharing via repositories. In the section below we take up one of the steps in the decision-making process but discuss the values broadly so as to provide the context within which we are considering them. When we come to the decision-making step-by-step process, we will again discuss these values specifically as they relate to the case study.

With respect to the issues discussed in this Domain, relevant *substantive values* include the following:

- *Autonomy/Liberty*: key considerations relating to autonomy and liberty include a research participant’s ability to make unforced decisions and choices about whether and how their data will be used in research. Such decisions hinge on a good understanding of material issues relating to the research. Hence, research participants should generally be advised if their data will be stored and used for other research, as well as what kinds of research that might include even if the data is anonymised.
- The *privacy* of individuals whose data is contained in research outputs made available in repositories is another consideration. Promises of anonymity may no longer be appropriate, especially if individual-level data is to be shared. Participants should also be made aware that withdrawal from future research may be difficult or impossible, once data has been shared. The maintenance of confidentiality is a related issue requiring consideration. The limited role of consent in respecting individuals’ privacy and confidentiality is important to consider, as consent itself does not protect from the risk of potential research participant re-identification.
- Promoting *public benefit/interest*, including making maximum use of data that has already been collected for research purposes, is another substantive value. A shift away from single use benefits arising from research is emerging in the scientific community, which now, more widely than ever before, acknowledges the significant benefits to the public of sharing data within and across disciplines, nationally, and globally.
- Another substantive value includes adhering to considerations of *justice* by:
  - Ensuring that suppliers of data are appropriately acknowledged and rewarded;
  - Establishing mechanisms to ensure that bona fide researchers from low- and middle-income countries are not systematically excluded from data access, which can occur in legislative and other governance efforts made to provide robust data protections;
  - Ensuring that the deposition of data, particularly from low- and middle-income countries, is done with participants’ knowledge that the data will be shared. The deposition of data in a repository, while promoting sharing in developed countries, could impede access by local researchers, which may also reduce the local benefits yielded from research conducted.

Key *procedural values* include transparency, accountability, and trustworthiness. These values relate both to processes adopted throughout data sharing but also to decisions regarding the development of repositories. An example of a data repository which has clearly articulated governance policies is *Brain-CODE* (www.braincode.ca). The clarity of these documents is important, as they increase transparency, which may also impact on accountability and trustworthiness.

- *Transparency*: the process of regulating access to sensitive data must be made transparent to research participants and also to researchers requesting access to data. The sharing of data for secondary uses has not been a requirement until recent years. If a data sharing plan is developed at the outset of the project, as is increasingly required, researchers will have a better understanding of the nature and requirements surrounding the sharing of their yet-to-be-collected data. Such planning at the inception of the research will enable researchers to more accurately determine the conditions under which data deposited in a chosen repository will be shared. Research participants will need to be given adequate accurate general information not only about the level of access others will likely have to data ultimately deposited in a repository but also who such individuals may be. They will need to be informed about the protections their data will enjoy as well as the potential risks of re-identification, even if remote. This information is readily available if researchers have already identified a data repository suited to the kind of data to be collected. Researchers, on the other hand, will need to have access to information regarding the processes adopted for decisions around access.
- *Accountability*: accountability relates to researchers whose claims and scientific findings should be available for scrutiny. This is particularly important in the context of big data research/projects, as it has been shown that the results of many medical studies cannot be replicated (Ioannidis 2005). Intertwined with this procedural value is the substantive value of harm minimisation to patients and the wider public when treatments are adopted into practice and when public policy is implemented based on non-reproducible research findings. Accountability also relates to researchers’ undertakings with research participants, especially where IPD is made available for re-use as well as accountability for inappropriate use/sharing.
- *Trustworthiness*: Researchers need to be confident that the systems used to store data are reliable both technically and administratively. As research participants are increasingly asked to have the research data they have contributed made available for re-use, they too will need to be confident that the organisations/entities hosting repositories as well as the systems and processes they employ will adequately protect their data when simply being stored and when being accessed.

## Case Study: Sharing Individual-Patient Level Data in Data Repositories

A clinician-researcher, Dr A, has completed a 2-year long city/state-wide, prospective observational study on the prevalence and risk factors for colonisation by antimicrobial drug-resistant bacteria in adult hospital inpatients. The study involved the collection of anterior nares (nose), groin, and rectal swabs and information on participants’ history of healthcare contact, recent antibiotic use, travel, as well as information on housing and occupation. Informed consent was obtained from all 2000 participants with the consent form stating that participants’ de-identified research data may be “shared for research and teaching purposes”. The approving IRB understood this to mean conferences, journal papers, workshops, and teaching activities, as deposition of data in a repository is not yet a required research practice at Dr A’s university. The university’s standard template for a data management plan, which Dr A had submitted, does not address the deposition of data into repositories.

Dr A intends to deposit the research data in an online discipline-specific data repository. Making the data accessible for future research has been strongly encouraged by the funder of the study and is mandated by the journal in which Dr A intends to publish his findings.

### Broad Considerations

This case exemplifies the difficulties that arise where the data sharing requirements of various entities clash, e.g. the journal mandates sharing of all research materials and products but the university has not yet formally considered data sharing as part of standard practice. It also highlights the fact that some stakeholders may be justifying their data sharing policies by appealing to certain values but inadvertently not attending to other important and relevant values. Thus, an IRB that prioritises harm minimisation of research participants over other values might have restrictive policies in relation to the disclosure of participant data, even if such data is de-identified. Such reluctance to embrace the sharing of de-identified data sets may result from concerns about appropriately adhering to privacy legislation. Conversely, scientific journals might be primarily concerned with the value of accountability, which would prompt them to support data sharing to allow for reproducibility.

Another broad consideration the case highlights is the importance of specifying what research data would have to be shared: would aggregate data suffice or is the sharing of IPD required? The sharing of aggregate data would avoid disclosing any information about individuals and, hence, would be less problematic. However, aggregate research data often does not allow for full reproducibility of results and is also less beneficial for future research use.

Researchers should anticipate, ideally at the stage of planning the research, that they will be required to share or deposit some or all research data upon publication or completion of a research project. Thus, they should consider incorporating requests for funding to support the potential additional costs involved in the deposition of research data into repositories (preparing the data for re-use can be time-consuming and expensive depending on the data). In addition, they should develop appropriate designs for the level of data sharing depending on the sensitivity of the data.

### Application of the Deliberative Balancing Approach

In this section, we apply the deliberative balancing approach that is introduced in Xafis et al. (2019) to the case study. The central question that we wish to consider is whether it would be appropriate for Dr A to upload the research data to an online research data repository.

1. *State the problem and distinguish the ethical issues that may arise from scientific, social, cultural, technical, and legal practices*

There are four issues to consider: 1. Dr A wants to publish the findings in a reputable journal but the journal requires him to make all underlying data (including de-identified IPD) available in a data repository. The study funders strongly encourage such practices. This puts him in an ethically challenging position. 2. Participants have consented to their data being used in anonymised form for future research—yet, in the era of big data, it is not clear whether such anonymity can be guaranteed. 3. It is unclear whether the statement in the consent documents “for further research and teaching purposes” adequately conveyed to the research participants that the data would be (potentially widely) shared through a data repository. 4. Technical issues to consider include the fact that securing anonymity may not be possible, especially when fine-grained individual participant data is involved and perhaps even more so when biological samples have been collected. Another technical issue is that it may be “impracticable” to re-contact the research participants to obtain their consent for the sharing of their data because of the number of participants involved (*n* = 2000) and because the study commenced 2 years ago.

Ethical issues include the following:

- Participants have consented to the sharing of their anonymised data for future research and teaching purposes—it might make a material difference in their decision making that such anonymity cannot be guaranteed. In fact, participants may have formed a legitimate expectation that their data will be completely secure and researchers may feel that they have to honour the expectation that they have generated.
- Although participants have agreed to the use of their anonymised data for future research, they may not be aware that their data will be stored in a repository in perpetuity and be made available to other researchers for purposes unrelated to the initial study.
- Re-use of data is in line with an ethos of open science. It enables reproducibility of results, thereby enhancing the integrity of the research. It also allows data to be re-used for new research purposes, which maximises the benefit of previously collected data.

2. *Identify the relevant values and potential conflicts among them*

The following are substantive and procedural values from the list of 16 Key Values that are listed in Xafis et al. (2019). Other values (from the list of 16) may be relevant as well, but those listed below are the ones that we deem to be most pertinent. Deciding which values must be considered can be challenging at first. To identify the values, we need to focus on the problem at hand, the ethical issues the case raises and also the obligations that arise as a result of our relationships with others. One of the central issues here is the assurances given and commitments made to the research participants as well as their expectations which flow on from these. The researcher is in a relationship of trust and owes respect to his/her research participants. Any deviation from what research participants expect as part of their involvement in the research process could undermine their trust in the researcher and the research community more broadly. On the other hand, making research data available has the potential to yield considerable benefits in relation to promoting research integrity and public benefit. Taking into account the issues listed in the first step of the application of the deliberative balancing approach as well as the need to respect persons and meet their expectations, we decided that the following values are most relevant:

- Substantive Values

Autonomy/Liberty: Participants’ autonomy is respected if conditions are created for self-determination with respect to medical data that is about themselves. Respect for autonomy is a key reason why the researcher obtains consent from participants, as it allows participants to make decisions about what they wish to be involved in. Such decisions should be free from external pressures if a research participant’s freedom to choose is to be supported.

Privacy: Even though the research data is said to be de-identified, in the age of big data we must acknowledge the potential for re-identification. Such re-identification would be a violation of privacy expectations in the sense that it violates participants’ freedom from unauthorised data activities involving information about themselves. If data did not identify individuals, they would likely be supportive of the inclusion of their data but, once re-identified, their privacy has been compromised.

Public Benefit: When considering public benefits, we need to bear in mind that these benefits are not identified as such by all.

- There is great public benefit arising from sharing this data, as the research findings can, if necessary, be validated. This not only contributes to the integrity of the research and findings but also potentially strengthens the relationship of trust between the research community and the public. There is generally great public support for scientific advances which can be accelerated at reduced costs and with greater efficiency when data is shared.
- An additional important consideration is who decides whose interests should be taken into consideration and what weight these interests should be given.
- Public and private interests do not always clash but consideration should be given to cases where private interests do not align with what are viewed as public benefits. In the case in question, it is impossible to determine if participants would be willing to assume the potential harm of re-identification of their data (even if this is unlikely).

Justice:

- The researcher wants to receive appropriate credit for his/her work; requiring the sharing of research data in a data repository that does not mandate appropriate attribution may result in an injustice to the researcher.
- Making the data available for re-use may produce public benefits but at the expense of those research participants who are opposed to their data being used beyond the current research project.
- The re-use of previously collected data has the potential to reduce burdens on the same groups of individuals if similar future research is to be conducted.

- Procedural Values

Trustworthiness:

- The research participants are likely to trust the researcher, as they have agreed to take part. Depositing their data in a repository against participants’ expectations could undermine this trust and have flow-on effects for the relationship of trust between individuals and the entire research community.
- Researchers are more willing to engage with systems and organisations they perceive as trustworthy. Where repositories are concerned, researchers need to have confidence in the systems employed, both technically and in relation to protections from exploitation or attacks. In addition, researchers will be more willing to deposit data if they trust the entities hosting the repositories.
- Research participants are known to be more willing for their data to be shared when they trust that great efforts are made to provide optimum protections for their data.

Transparency:

- The deposition of data in a data repository and its re-use have not been clearly communicated to participants, who are likely to be unaware that such data sharing is rapidly becoming the norm.
- Transparent governance structures require repository curators to comprehensively and openly state the conditions of access to those intending to deposit data so that data owners can make appropriate decisions regarding the level of access they believe is appropriate for their data and research materials.

Accountability:

- By sharing data, researchers become more accountable for the findings they publish, as it is possible for others to scrutinise their work.
- The researcher is accountable to his research participants who expect him/her to honour assurances given during the consenting process.

3. *Identify actions that could be taken and the values underlying these*

Several courses of action may be pursued. The most salient ones are:

- Open Access Sharing

Open sharing of IPD is considered valuable for the generation and testing of new hypotheses and the conduct of meta-analyses. Open sharing would strongly prioritise public benefits and is foundational to promoting accountability in the research enterprise.

- Sharing Data on a Case-by-Case Basis

The sharing of data can be done on a case-by-case basis which would involve a researcher identifying the research Dr A has done and contacting him for the underlying data. This form of data sharing relies on other researchers being familiar with the research someone has conducted, as the data is not publicly listed anywhere. Therefore, the underlying data cannot be discovered by researchers accessing repositories to identify suitable data for further analysis. Features unique to this kind of data sharing include the following:

- Dr A would be required to establish the credentials of the requestors
- Dr A would need to establish the conditions under which the data could be used
- The above would impose additional responsibilities on him and could be time-consuming
- Additional time may be required for different formatting and extraction requirements depending on the request.

- Restricted Access

Depositing the research data (including de-identified IPD) in a well-governed repository with restricted access is another option. This would reduce the risks to research participants for several reasons:

- Data has been de-identified
- Access is restricted to bona fide researchers only
- Repository administrators have clear articulation of the responsibilities and expectations that researchers who access data have for its responsible re-use in research, and
- Several data security mechanisms are likely to be in place but, as these vary depending on the repository, they would need to be checked in advance.

In combination, these features may suffice to provide adequate and reasonable data protections. Such requirements point to the weight given to ensuring that the privacy and confidentiality of individual participants are protected and that participants are not inadvertently harmed in the process of researchers sharing data for broader public benefits. Public benefit relating to the re-use of data would be supported by this course of action and aspects of the values of transparency and accountability would also be promoted.

- Provide/Deposit Aggregate Data Only

Dr A could agree to provide aggregated data only, explaining to the journal that explicit consent for the deposition of IPD had not been requested at the start of the project. Dr A would be acting in accordance with his research participants’ expectations for respect, trustworthiness, and accountability but would potentially be viewed as not being transparent in his research practices, as the IPD would not be available for scrutiny by others.

- Contact the Research Participants

Dr A could make efforts to re-contact research participants to explain the nature of the issue which would include clarifications of the following: new requirements to deposit all data in a repository in a de-identified form; efforts to ensure data cannot be re-identified but that this could not be guaranteed; re-use of data by bona fide researchers only. The provision of such information would demonstrate Dr A’s respect for participants and would show that the researcher is transparent about his intention to make available the data for future research.

4. *In light of the values and context, weigh up the relative ethical merit of the different options*

- Open Sharing (Option 1)

- Open sharing of the research data would potentially yield the greatest public benefit, as other researchers would have access to IPD.
- Open sharing might create unintentional harms for the research participants, as the potential for re-identification could be greater as a result of it being available to anyone wishing to access it.
- Even if participants did not find out that the data had been shared against their expectations, it could be argued that they have suffered a harm because they were not shown due respect as research participants.
- If the research participants in this study did find out about the sharing of the data for other research via a repository, it could undermine the established relationship of trust between the public and the research community at large.

- Sharing Data on a Case-by-Case Basis (Option 2)

- This kind of data sharing would not yield the greatest public benefit because the data would not be discoverable.
- Data preservation could not be guaranteed.
- Individual requests would impose additional obligations on a researcher including, for example, adequately checking the requestor’s credentials and keeping a record of the requests made.
- The handling of the data may differ from request to request potentially raising concerns about transparency of processes and the protections provided to research participants.

- Restricted Access (Option 3)

- Uploading de-identified IPD (and associated metadata) and imposing restricted access on the data affords additional protections, particularly when specific requirements exist for data requestors to acknowledge that they have significant obligations in relation to the way the data will be used by them.
- The credentials of the requestors are more likely to be scrutinised where restricted access data is concerned. This improves transparency around who is using the data and for what purpose.
- The data will be available for future uses and may contribute to additional public benefits.
- Even though specific clarifications will not have been made to research participants regarding the deposition of their de-identified data, the researcher has made efforts to provide greater protections thus potentially minimising harms these research participants may otherwise have suffered.

- Provide/Deposit Aggregate Data Only (Option 4)

- The deposition of aggregated data only would be in line with what research participants would be expecting. This would provide the greatest protections for participants and would promote the relationship of trust established.
- Not re-using valuable IPD would significantly reduce the research yield which would have promoted potentially important public benefits.
- Not making IPD available would impede the reproducibility of results.
- It is likely that the journal would not accept such a justification or course of action. This would place the researcher in a difficult position as he has substantial obligations as a researcher to publish the findings.
- If the journal of first choice does not agree to have aggregate data deposited in a repository, the research may be published in a less prestigious or relevant journal, which could impact on the wider dissemination of findings.
- Even if Dr A decides not to upload the IPD to a data repository, he may consider sharing the data on a case-by-case basis, when contacted by other researchers. This would allow the researcher to retain tight control over access to the data—but there is the risk that a researcher may use this power arbitrarily and, for instance, share the data only when he is granted co-authorship on future publications originating from the use of the data. The same demands for co-authorship can also arise where data is requested via a repository.

- Contact the Research Participants (Option 5)

- Contacting all 2000 research participants (provided the contact details are still available to the researchers) would be an acknowledgment of the importance of respecting their wishes as well as adhering to expectations around transparency and accountability.
- As this activity was unanticipated and the research study has been completed, there may not be sufficient funds to dedicate to re-contacting participants. Furthermore, the research project commenced 2 years ago. There is a likelihood that some individuals may no longer be contactable.
- Contacting the research participants could impact on the researcher’s ability to deposit the data of those who are not willing to contribute their data to the repository collection. This would very likely not be acceptable to the journal, as the dataset would be incomplete.

5. *Select the option that has the strongest ethical weight attached to it*

- Option 3—Depositing the Research Data in a Repository with Restricted Access

It is usually impossible to satisfy all values that relate to a particular ethical concern but carefully considering the specific circumstances helps in weighing them against each other and identifying the option that can satisfy the most central values to the greatest degree. Here, it seems impracticable, and perhaps problematic, to re-contact the participants but we must ensure that their data and privacy are well protected. Such choices would promote the trust between the research community and publics and would provide evidence of the researcher’s respect for research participants, as their welfare is a central consideration. However, we must also bear in mind important considerations beyond the research participants themselves.

A preferable option is to deposit the research data in a repository with restricted access. Dr A should identify a data repository with robust governance structures, which allows researchers to set the access level and which conducts appropriate screening of data requestors. Such screening may involve verifying affiliations, qualifications, and requiring a commitment they will not share the data with others or attempt to re-identify individuals. As previously noted, the specific features of the data, such as the level of sensitivity and the extent to which it can be meaningfully de-identified, will vary and will determine the level of access others could or should have to the data deposited in a data repository. Levels of access are often determined by each repository but researchers can also have input into this depending on the repository. Each repository makes governance documents available to researchers and other users and the level of detail in these documents reveals, to a large extent, the weight the repository places on many of the values discussed in this section.

This option attempts to strike a balance between numerous values identified as underlying research and the increasing requirement to share data. On the one hand, it shows consideration for participants’ privacy and confidentiality by seeking to increase the technical and governance protections which all aim to reduce the potential for harm that might otherwise arise. On the other hand, restricted access to the IPD would enable other researchers to gain greater value from the data and to develop research projects that could further explore the area in question without engaging new research participants. Such research would require fewer funds, which, cumulatively, is of great benefit to the general public.

6. *Communicate the action to be taken to all relevant stakeholders*

Dr A must now contact the IRB and the funding body to advise them of the data repository he has selected. If his research is referenced on his university website, he could indicate there which restricted access data repository he has deposited the data in. This would increase discoverability.

## Conclusion

This paper discussed the *Domain of Openness in Big Data and Data Repositories*. It presented issues that arise in open data sharing in the context of big data and provided insight into the nature of data repositories. The paper provided a case study which allowed us to firstly consider in a broader sense some values identified as being relevant. We then used the Framework by first identifying the values related to the case in question and then by applying the step-by-step decision-making process previously described (Xafis et al. 2019). Where necessary, explanations were given to elucidate further how selections and prioritisations were made. The recommended option was justified but further justification could be given by referring to the reasons why the other options were discounted.

## Footnotes

1 “Open Data” has been defined as “data that can be freely used, re-used and redistributed by anyone—subject only, at most, to the requirement to attribute and sharealike” with the *Open Definition* (https://opendefinition.org/od/2.1/en/) giving full details of what “openness” entails.

2 “Confidentiality” refers to one’s duty not to disclose information shared and the resulting understanding between two or more parties about how such information should be treated.

3 N.B. we are not using the term “sensitive data” in the legal sense used in some jurisdictions.
